# Substitutes for Bear Bile for the Treatment of Liver Diseases: Research Progress and Future Perspective

**DOI:** 10.1155/2016/4305074

**Published:** 2016-03-21

**Authors:** Sha Li, Hor Yue Tan, Ning Wang, Ming Hong, Lei Li, Fan Cheung, Yibin Feng

**Affiliations:** School of Chinese Medicine, Li Ka Shing Faculty of Medicine, The University of Hong Kong, Pokfulam, Hong Kong

## Abstract

Bear bile has been a well-known Chinese medicine for thousands of years. Because of the endangered species protection, the concept on substitutes for bear bile was proposed decades ago. Based on their chemical composition and pharmacologic actions, artificial bear bile, bile from other animals, synthetic compounds, and medicinal plants may be the promising candidates to replace bear bile for the similar therapeutic purpose. Accumulating research evidence has indicated that these potential substitutes for bear bile have displayed the same therapeutic effects as bear bile. However, stopping the use of bear bile is a challenging task. In this review, we extensively searched PubMed and CNKI for literatures, focusing on comparative studies between bear bile and its substitutes for the treatment of liver diseases. Recent research progress in potential substitutes for bear bile in the last decade is summarized, and a strategy for the use of substitutes for bear bile is discussed carefully.

## 1. Introduction

Bear bile is the dried gallbladder bile collected from the black bear (*Selenarctos thibetanus*), the brown bear (*Ursus arctos*), or other species of Ursidae, under the category of animal drugs in Traditional Chinese Medicine (TCM), and has been used in TCM clinical practice for thousands of years [1–3]. Among the classic prescriptions of TCM, there are 396 kinds of prescriptions containing bear bile constitute [4]. In the point of TCM view, bear bile is a cold medicine and is bitter in flavor and cool in nature, and it is entering meridian of liver, gallbladder, and heart, so it could clear heat, relieve toxin, clear away liver fire, and stop endogenous wind [5, 6]. In recent decades, modern pharmacological studies also claimed that bear bile has a wide range of pharmacological actions, including hepatoprotection and antibacterial, antiviral, anti-inflammation, antigallstones, hypolipidemic, and some other effects [7–10]. In TCM clinical practice, bear bile was used in fever fighting, detoxification, and reduction of inflammation, swelling, and pain [4, 5]. Particularly, it was widely used for curing a variety of liver diseases, such as fibrosis, biliary cirrhosis, and even liver cancer [6]. Bear bile shows strikingly abilities to reduce liver fire and liver heat, which refer to the pathological phenomenon within the liver from the point of Chinese medicine view.

However, the application of bear bile has drawn substantial concerns and controversy from worldwide public, media, and animal rights folks. The first concern is that the extensive consumption of bear bile in China and other Asian countries has made bears become endangered species. Although bears are listed in the Convention on International Trade in Endangered Species of Wild Fauna and Flora (CITES), the illegal abuse of innocent bear for huge profits worldwide is continuing [3, 11–13]. The second concern is the extreme cruel and inhuman method to extract bile from living bear. The extraction method named “Free-dripping Fistula Technique” by inserting a catheter into the bear gallbladders to drain the bile daily was applied in thousands of bear bile extraction factories [14, 15]. It once was thought to be a great technological progress from “kill the bear to get the bile” to “drain bile from living bear.” But the extraction of bile from living bear is such an unbearable torture, which does not kill the bear in an instant but will render it a pain worse than death. The bears would suffer tremendous mental stress and physical trauma for decades and result in illnesses and chronic infections caused by the presence of foreign bodies and wounds [4, 16]. The third concern is the lasting controversy of whether the use of bear bile is unsubstitutive as it was claimed. Certain TCM physicians declare that there is no alternative product which can substitute bear bile in terms of its integrative effect. However, by a great number of modern pharmacological and chemical investigations, increasing drugs and compounds are considered to be the substitute for bear bile [14, 15, 17].

Actually, the usage of bear bile is a complex issue concerned with culture, economy, and even politics. The reasons underlying the issue may merely be caused by not only the imperfection of legal protection against bear but also the biased belief of people that expensive and uncommon drugs are associated with better effects. Facing these challenges, in recent years, accumulative systematic researches on seeking substitute of bear bile have been carried out by many research groups. It might be a struggling objective to replace the bear bile by artificial material with the same composition, but it is possible to provide scientific evidences of the comparable curative effect of potential substitute [11, 12]. Artificial bear bile, bile from other animals, synthetic compounds identified from bear bile, and some herbs from TCM are regarded as the most promising substitutive materials for bear bile [4]. A rational way to protect the endangered bears and stop the abuse of bear bile requests is not only comprehensive research in definite composition and pharmacological actions of bear bile but also comparative research in treatment efficiency of the probable substitute [15, 18]. In this review, the research progress of bear bile and its potential substitute in last decade will be updated, and predominant attention will focus on liver diseases. Furthermore, challenge ahead for the realization of substitute of bear bile will be discussed thoroughly.

## 2. Recent Research Progress in Bear Bile

### 2.1. Composition of Bear Bile

**Figure 1:**
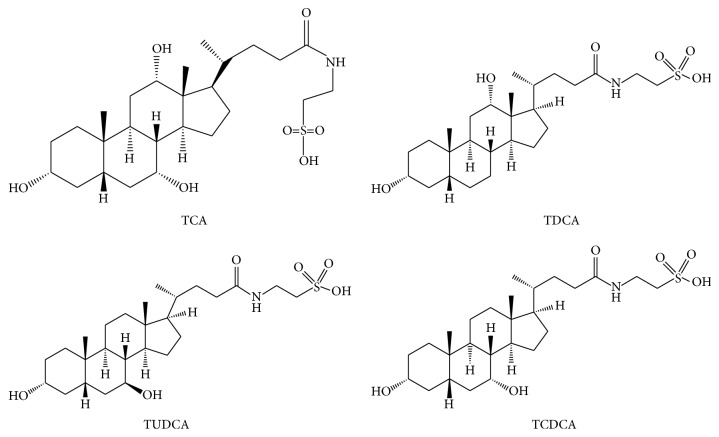
The chemical structure of bile acids in bear bile.

During past decades, extensive research has been performed to qualify and quantify the composition of bear bile, especially the total and individual bile acids. Modern chemical research indicated that bear bile was composed primarily of bile acids, phospholipids, cholesterol, bile pigments, proteins, and inorganic salts [19]. Among these components, bile acids may play a major role in therapeutic action for bear bile. Bile acids are synthesized from cholesterol by the hepatocytes and are concentrated and stored in the gallbladder [7, 11–13]. All the bile acids in bear bile are in the form of taurine conjugate, including taurocholic acids (TCA), tauroursodeoxycholic acids (TUDCA), taurodeoxycholic acids (TDCA), and taurochenodeoxycholic acids (TCDCA), which are shown in Figure 1. These conjugated bile acids are formed through the conjugation of amino acid taurine with each of the corresponding free bile acids. In a survey study, the contents of two major constituents, TUDCA and TCDCA, in 8 commercial batches of bear bile were detected as 39.04 ± 7.83% and 36.10 ± 9.42%, respectively [20]. The average concentrations of TUDCA, TCDCA, and TCA in 93 Ursidae bile salts were 3087.8 ± 1626 *μ*g/mL, 1968 ± 678.1 *μ*g/mL, and 212.6 ± 154.1 *μ*g/mL, respectively [21]. Of note, it is believed that the relative high level of TUDCA and TCDCA makes bear bile different from biles derived from other animals [4, 15, 19].

However, the composition of bear bile is not constant; instead, it changed by a variety of factors, such as existing style, species, physical state, and season. It has been reported decades ago that a remarkable reduction of TCA and a dramatic increase of TUDCA and TCDCA were detected in the bile which is originated from farmed bears [19]. In terms of the differences between species, it was found that the content of TCA in North American and polar bears was higher than that in Asiatic bears [21]. The seasonal change of the composition in bear bile was also observed. In a study, the composition contents of gallbladder from 8 active and 14 dormant black bears were analyzed by HPLC. The proportion of TUDCA to the sum of the TUDCA, TCDCA, and TCA was decreased and the concentrations of cholesterol, phospholipids, and metals including calcium, magnesium, zinc, and copper were increased. The results indicated that a striking decrease of metabolic activity in the gut flora is kind of adaptation to metabolic stability of the dormant bear [20–22]. In practice, the variation of bile bear composition is crucial and worth noting, which is of great importance to identify the quality of bile.

Furthermore, some new compounds were identified and isolated in bear biles recently. Two new bile acids, tauroselocholic acid and tauroansocholic acid, and a new natural bile acid, cygnocholic acid, were isolated from bear bile powder [23, 24]. Several flavone compounds were also identified in bear bile, including 4′,7-dihydroxyisoflavone, 4′,7-dihydroxy-6-methoxyisoflavone, 4′,6,7-trihydroxyisoflavone, and 4′-methoxy-7-hydroxyisoflavone [22]. The identification and isolation of new compounds may provide scientific basis to further investigate their pharmacological actions and mechanisms.

### 2.2. Pharmacologic Effects and Clinic Application

Modern pharmacological studies confirmed that bear bile powder has a variety of physiological activities, such as antibacterial, antipyretic, sedative, anti-inflammatory, antispasmodic, anticonvulsants, antistress, dissolving gallstones, hypoglycemic, and antitumor effects. It also shows some effects on the cardiovascular system, which can lower blood glucose and lipids, relax blood vessels, and inhibit thrombosis [25–30]. In TCM clinical practice, bear bile was used in fever fighting for seasonal febrile disease, detoxification, heat-toxin syndrome, and reduction of inflammation and pain. Besides traditional use, the modern application of bear bile was spread widely to many modern diseases based on traditional indications and pharmacological effects. Clinically, modern medicine extensively applies bear bile to eye disease, hemorrhoids and gallstones, bile reflux gastritis, viral hepatitis, gallstone pancreatitis, fatty and alcoholic liver disease, drug-induced hepatitis, and other cholestatic liver diseases. In terms of eye disease induced by overbalance of liver fire and heat-toxin, bear bile shows remarkable therapeutic effect.* Xiongdan Eyedrops* made by bear bile is a famous drug to treat eye disease. From Chinese medicine perspective, it is believed that the eyes act as the window of the liver; therefore, the specific effect of bear bile on eye disease also implies its outstanding application in liver disease.

## 3. Bear Bile in Liver Diseases

As bile is predominantly produced by hepatocytes to digest fat, it plays an important physiological and pathophysiological role in the body. The lack or disorder of bile, especially for bile acids and bile salts, causes fat digestion problems and related hepatobiliary diseases. It is believed that exogenous administration of animal biles to supply bile compositions could attenuate these diseases. Clinically, bear bile is widely used not only for hepatobiliary diseases but also for other liver diseases, such as viral hepatitis, fibrosis, or liver cancer [12–14, 16, 31–36]. In traditional clinical practice, bear bile was used in the cure of overabundance of liver fire and redness of eyes due to liver heat. Although it indeed shows positive therapeutic effect to a certain extent, the mechanisms concerning how it works still have not been fully elaborated. Generally, people attribute the hepatoprotective effects of bear bile to its immunity enhancement and microenvironment regulation abilities [7, 11, 12].

The comprehensive metabolomics profiling and pathways in animal models with hepatitis C virus (HCV), a leading cause of liver disease worldwide, have been investigated in a study. It was found that the proteins of bear bile powder, a widely used anti-HCV drug, could relieve HCV infection partially by modulating the perturbed pathway, but the specific mechanism and pathways are not clear until now [30]. With regard to alcoholic injury, bear bile additionally showed protective effects [31]. Protective effect of bear bile powder on alcohol-induced oxidative damage in mouse primary cultured hepatocytes was also demonstrated [31]. It has also been indicated that bear bile possesses inhibitory efficacy against the suppressive immune activity induced by hepatic stellate cells [32, 33]. The immunosuppression activity induced by hepatic stellate cells in the injured liver plays a vital role in the pathogenesis of liver disease, including fibrosis and hepatocellular cancer. Bear bile could decrease the population of hepatic stellate cells, regulate the surface molecules, and thus affect the related cytokine secretion. Furthermore, hepatic stellate cells isolated from the mice treated with bear bile can promote the proliferation of T cell, decrease regulatory T cells, and enhance the activation of T cells, which means bear bile may improve immune response in liver disease [34–36]. The research of our group also proved that bear bile derived from Asiatic black bear showed effects at a certain level on the fibrosis induced by different factors, including CCl_4_, bile duct ligation, and alcohol [15].

In addition to the above liver diseases, the effect of bear bile on liver cancer has been broadly studied in diverse models. It was demonstrated that bear bile powder could inhibit the growth of HepG2 hepatocellular cancer cells in a dose-dependent manner through activation of caspase-9 and caspase-3 and thus leads to cell apoptosis [37]. In a nude mice hepatocellular carcinoma xenograft model, it was found that bear bile could induce the apoptosis of cancer cells and inhibit their proliferation by upregulating the expression of Bax and inhibiting the protein expression of Bcl-2, PCNA, p-STAT3, CDK4, and cyclin D1 [38]. In terms of liver cancer induced by diethylnitrosamine, bear bile could prevent the progress of hepatocarcinoma in rat models, which may be related to its inactivation of hepatic stellate cells, and thereby alleviate fibrosis and cirrhosis [39]. Additionally, the combined treatment of bear bile powder and cyclophosphamide shows protective effect on colorectal cancer liver metastasis by increasing immunity and regulating tumor microenvironment, particularly, by reducing inflammatory cell infiltration [40].

## 4. Substitute of Bear Bile

Actually, the concept of substitute of bear bile has been raised for decades since people realized the miserable situation of bear bile. Looking for substitute of bear bile means finding a kind of material which does not originate from endangered species and possesses similar therapeutic effects to those of bear bile to replace the clinical use of bear bile [15]. A basic principle for substitute of bear bile is the similar chemical or pharmacological profiles. In this respect, four kinds of substitutes for bear bile are considered: (1) artificial bear bile, (2) synthetic compounds, (3) other animal biles, and (4) medicinal plants [4, 11, 12]. In addition to fit Chinese medicine view, modern systematic research has to be performed to provide scientific evidences for an alternative of bear bile.

### 4.1. Artificial Bear Bile

Artificial bear bile is the counterfeit of natural bear bile, which contains almost similar compositions and structures of various synthetic or natural bile acids, amino acids, minerals, cholesterol, and bilirubin to the natural ones. In fact, artificial bear bile contains almost all the active ingredients contained in high-quality natural bear bile, and the content is very close [41]. TUDCA, which is a specific compound that has been only discovered in bear bile so far and does not appear in the other animal bile samples, was also detected in artificial bear bile, which reduces apoptosis by modulation of AP-1 and shows anticholestatic effect [42–44]. In certain cases, the content and stability of the active compounds are even high and better than drainage bear bile. The similar chemical composition constructs the basis of its comparable pharmacologic effects to those of bear bile [4, 15]. Meanwhile, research has pointed out that artificial bear bile has shown similar efficacy to natural bear bile, such as antibacterial, anti-inflammatory, and antispasmodic effects [41]. Hundreds of clinical trial results indicated that, in the treatment of artificial bear bile on acute tonsillitis and anger hyperactivity hypertension, artificial and natural bear bile show no significant difference in efficacy.

Although artificial bear bile displays strong competitiveness and potential as substitute of bear bile, totally replacing the natural and drainage bear bile with artificial bear bile is a long way to go. Despite challenge ahead, the successful application of some substitutes of traditional expensive Chinese medicine drugs, such as artificial tiger bone used to replace tiger bone, still leads to enthusiasm for the possibility of replacing bear bile with artificial bear bile.

### 4.2. Synthetic Compounds

Among the chemical components in bear bile, bile acids were regarded as the main effective components; among the bile acids, UDCA and TUDCA have attracted most attentions. As a matter of fact, UDCA, which is named from the root word for bear,* urso*, was first discovered in bear bile in 1900s. Of note, in humans, UDCA is a second bile acid, which is the metabolite of primary bile acid by intestinal microbiota; whereas in bears, it is made directly from cholesterol by hepatocytes and then conjugates with taurin to form TUDCA. Theoretically, it is believed that UDCA exists in bear bile with relatively low content; however, several studies that aimed to determine the composition of bear bile revealed that UDCA was not detected by modern chromatography technique [19, 45], which might be due to the limit of detection or the status of bear. This controversy is of great importance and is worth noting because UDCA shows remarkable pharmacologic action and was traditionally considered to be the primary bioactive component of bear bile.

UDCA, which is hydrophilic unlike other common bile salts, significantly relieves liver damage in the setting of cholestasis [46–49]. In 1950s, it was synthesized successfully and adopted to treat liver diseases clinically [50]. Clinical studies have confirmed the beneficial effects of UDCA in a broad variety of cholestatic liver diseases, including primary biliary cirrhosis, pediatric cholestatic disorders, primary sclerosing cholangitis, and drug-induced cholestasis [51–55]. It is assumed that UDCA replaces the more detergent and potent endogenous dihydroxy bile acids within the bile acid pool, principally by inhibiting their active absorption from the intestine [56, 57]. In addition, UDCA also promotes membrane insertion of transporters for biliary lipid at the level of the hepatocyte canaliculus [48, 50]. High-dose UDCA (28–35 mg/kg per day) was administered to patients with nonalcoholic steatohepatitis; the normalized aminotransferase levels, reduced serum fibrosis marker, and improved markers of glycemic control and insulin resistance were observed, which revealed that UDCA is an ideal drug for nonalcoholic steatohepatitis [57, 58]. For liver injury induced by drug, UDCA also shows respectable therapeutic effects [59, 60]. Additionally, with regard to hepatectomized liver transplantation model and hepatocellular carcinoma, UDCA could relieve the hepatic damage [61–63]. Of note, UDCA is the only drug for patients with primary biliary cirrhosis which is approved by the Federal Drug Administration. Since UDCA has not been detected in bear bile by modern studies whereas it has been confirmed that it possesses outstanding pharmacological functions, the replacing of bear bile in liver disease by synthesized UDCA is rational and promising. The synthesis technology successfully meets the demands of large quantities of UDCA for medicine use [64].

TUDCA, the specific bile acid in bear bile, is also a hydrophilic bile acid that is synthesized in the conjugation pathway of UDCA, which acts as bile secretagogues and immunomodulators [65, 66]. In addition to cholestatic liver diseases, TUDCA shows hepatoprotective effects during long-term ethanol feeding and ischemia-reperfusion injury [67, 68]. It can prevent apoptosis in hepatic and nonhepatic cells induced by various factors, such as hydrophobic bile acids, Fas ligand, transforming growth factor *β*1, and alcohol [68, 69]. The mechanisms involved include improving mitochondrial function and integrity and interactions with NF*κ*B pathways [69]. The synthesized TUDCA, which has been produced and used as drug and supplement, to say the least, partially represents the effective component of pharmacological action of bear bile. Therefore, TUDCA could replace the use of bear bile in many contexts.

In addition to UDCA and TUDCA, several other bile acids are extensively studied for their pharmacological effects. DCA, one of the secondary and free bile acids, are metabolic byproducts of CDCA by intestinal bacteria. The main function of DCA is to dissolve gallstones. The generation of gallstones is due to oversaturation of cholesterol in the bile, forming a rock after precipitation, while DCA could inhibit reabsorption of cholesterol in the intestine and blockage of cholesterol to bile secretion, thereby reducing the concentration of cholesterol in the bile [4, 6, 19]. It has been indicated that CDCA, an isomer of UDCA, could improve the composition of bile and increase the solubility of cholesterol with less toxic side effects [70]. It was also found that it could upregulate the expression of fibroblast growth factor 21 in liver of C57/BL6 mice, resulting in a decrease in plasma triglyceride, cholesterol, and blood glucose [71]. A study performed by our group showed that the cytotoxic activity of DCA, CDCA, and TCDCA on liver cancer cells MHCC97-L is better than that of bear bile, which gives a hint that they might be an alternative to bear bile in prevention of liver cancer and as a therapy for the same [19]. With regard to HDCA, it is a kind of specific deoxycholate extracted from pig, whose structure is slightly different from that of UDCA. The function of HDCA is close to that of UDCA, showing significant ability to lower blood cholesterol [6].

As mentioned before, accumulating scientific evidences demonstrated that these bile acids are suitable to replace the usage of bear bile in medicine, especially in terms of chronic liver disease. In fact, the successful synthetic technique of these DCA-based acids not only meets the need in quantity but also provides more stable quality of drugs. As of today, most of bear bile products are made by biles extracted from living bears, which always get infected from the open wound [4]. The replacement of synthetic bile acid compounds might effectively solve these drawbacks of bear bile, including unstable composition and poor quality control. However, concern might be raised that single compound could not exert integrative effects, which is pursued and highlighted in Chinese medicine. The combination of several active components could be considered in the future.

### 4.3. Biles from Other Animals

In TCM, it is believed that drugs with similar efficacy and taste generally have a certain similarity of pharmacological properties, which gives a hint that they might be used alternatively [4, 72]. For example, buffalo horn clinically is often used instead of rhinoceros horn, thus solving the contradiction between endangered rhino protection and medicinal usage. In the same respect, other animal biles have highly possible potential to be the substitutes of bear bile. Extensive studies have been conducted to investigate the differences and similarities between bear bile and other animal biles in terms of composition and pharmacological actions.

Plenty of studies have been carried out to compare the compositions of biles derived from other animals with bear bile; particularly attention was paid to bile acid due to its vital role in pharmacological activities. The bile acid compositions of several animals, including snake, pig, cattle, chicken, and rabbit, were preliminarily studied to compare with bear biles [19, 73]. Remarkably, the variants of chemical composition of the animal bile were observed. Pig bile contained a great deal of UDCA, whose taurine conjugated form is TUDCA. Snake bile acid was primarily comprised of TCA and rabbit bile contained a great deal of glycodeoxycholic acid (GDCA), whereas cattle bile acid mainly consisted of DCA, CDCA, and TCDCA, a series of DCA-based chemicals. The presence of various primary bile salts, which change into bile acids in acidic conditions in common animal biles recorded in traditional Chinese medicine, was listed in Table 1 [6, 19, 72–75]. As seen from Table 1, many other animal biles contain TC, TDC, and TCDC, as bear bile does. TUDC, as specific compound in bear bile, is also present in mouse bile, which is worth to be further studied [73].

Meanwhile, in addition to the comparison of compositions of bile acids, increasing comparative researches to study the pharmacologic actions of different animal biles have been carried out. Many pharmacologic actions of the rabbit bile and cattle bile were similar to those of bear bile and even stronger than those of bear bile in terms of some effects, such as antihistaminic and antitussive abilities [6, 76, 77]. With regard to the relief of hepatocyte injury induced by CCl_4_, the therapeutic effect of rabbit bile was as good as that of bear bile. For pig bile, it was reported that both bear bile and pig bile possess comparable bioactivities, such as anti-inflammatory and analgesic effects [4, 6]. Furthermore, it has been indicated that pig bile possesses antiallergic effect on delayed-type hypersensitivity, a feature shared with bear bile but not with cattle, which might be due to the presence of UDCA. The cytotoxic effect of bile from bear species and other several animals on the hepatocellular carcinoma cell line MHCC97-L was tested using the MTT assay in our group. Our results indicated that biles from pig and cattle showed potent cytotoxic effects in MHCC97-L cells, which were notably better than that of bear bile [15]. These several kinds of biles were generally regarded as the most promising substitutes of bear bile based on the constitute compositions and pharmacological effects.

Since the function and chemical composition of many other biles have certain similarity to bear bile, bile acids derived from other animals provide great prospect for the development of bear bile substitutes. Certainly, it is not acceptable to transfer the miserable destiny from bear to any other animals. In fact, the substitute for bear bile from other animals remains controversial concerning the animal welfare and animal rights. In China, for the most livestock such as pig, cattle, and duck, their internal organs including gallbladders will be abandoned after slaughter [6]. In this case, using biles from livestock as alternatives of bear bile not only protects endangered species and prevents crucial extraction but also avoids the waste of resources.

### 4.4. Traditional Medicinal Plants

Traditional Chinese medicinal plants are abundant resources to look for alternatives for bear bile. Actually, searching for substitutes of rare and expensive traditional drugs in Chinese medicinal plants has a long history and many successful cases have been created, for example, Dangshen (*Codonopsis pilosula* (Franch.) Nannf.) and Xiyangshen (*Panax quinquefolius* L.) as substitutes for Renshen (*Panax ginseng* C. A. Mey.). The declaration that “bear bile cannot be replaced” is made on the basis of integrative effects highlighted in Chinese medicine; however, alternative Chinese medicinal plants have the potential to reach integrative treatment efficacy from Chinese medicine view. In TCM, bear bile was characterized under the category of clearing heat and detoxification drugs [6, 7, 11, 12]. Therefore, those Chinese herbs under the same category are the first objects for consideration to be substitutes of bear bile [78]. Chinese herbs with the efficacy of clearing heat and detoxification are listed in our previous published paper [4]. For example,* Scutellaria baicalensis* Georgi has been studied as the herbal alternatives to bear bile, which shows effects on IL-6 promoter and CYP3A4 activities [79]. But it is regrettable that until now no study has been carried out to systemically compare their pharmacological effects with that of bear bile by modern scientific research methodology.

Among the promising plant alternatives for bear bile,* Coptis*, “Huanglian” in Chinese, is an outstanding candidate. Seen from TCM view, both bear bile and* Coptis* are under the category of clearing heat and bitter taste; moreover, both of them could detoxify and purify liver and improve eyesight according to Chinese medicine theories. In TCM clinical practice, they have similar clinical indications and are often used to treat liver diseases [80, 81]. In our group, extensive researches have been performed on the pharmacological actions of* Coptis* and its main bioactive compound berberine. By our past researches, it could be concluded that* Coptis* and berberine possess convincing and satisfactory effects on the treatment of various liver diseases, including acute and chronic liver injury induced by CCl_4_, alcohol, bile duct ligation, fibrosis, and liver cancer [82–86]. Strikingly, a comparative study to investigate the therapeutic effects of bear bile and* Coptis* extract on liver fibrosis in rats has been conducted by our group. Apart from that, the hepatoprotective actions of their main bioactive compounds, TUDCA and berberine, respectively, are also compared. The results we obtained are interesting and not disappointing. We found that the effects of berberine and* Coptidis Rhizoma* to treat fibrosis are much better than that of bear bile in the same experimental condition. It was shown that berberine and* Coptidis Rhizoma* could upregulate the activity of superoxide dismutase (SOD), reducing oxidative stress. The reduced serum total bilirubin levels indicated that berberine was able to excrete the bilious product and prevent the hepatocyte injury [15]. These results throw light on using berberine as substitute of bear bile to treat diverse liver diseases including cholestatic and noncholestatic liver fibrosis. But the disadvantage of plants substitution also exists. Substitution with totally different compositions makes it hard to explain the similar medicinal effects by modern research.

## 5. Challenge Ahead and Strategy

### 5.1. Scientific Research

Although accumulating researches have shown positive results for the development of substitute of bear bile, more convincing data from modern basic research and clinical study are required [15]. Regrettably, there are only few systematic and scientific researches which had been performed to compare the medicinal effects between potential substitutes and bear biles. For instance, the medicinal effects of UDCA and TUDCA, which are treated as the primary effective components in bear bile, are supposed to be compared with bear bile in the same condition. For basic research, apart from effect, the mechanism of bear bile and its main bioactive compound should also be investigated. By knowing how it works* in vivo* to exert medicinal effect, more targeted substitutes could be noted and attempted. With regard to clinical study, an indispensable part to realize the replacement of bear bile, trials with large-scale patients on the medicinal effects of substitute of bear bile should be designed and conducted. People who insist on their belief that bear bile could not be replaced with Chinese medicine are also supposed to provide scientific evidences to prove it. The debate of bear bile and substitute might be solved by rational research data [4, 6]. With only sufficient scientific evidences provided, people might change their traditional mind and have faith in bear bile substitute. Besides the interest and awareness of endangered species protection, sufficient financial support is vital to carry out these studies.

### 5.2. Government and Policy

Government, which makes the policy, plays an extremely important role in the development of bear bile substitute. Actually, with regard to Chinese government, whether bear bile should be used or not is a dilemma. On one hand, bear bile is a part of Chinese medicine and is also a part of traditional national culture, which is supposed to be preserved and cherished. On the other hand, there was constant pressure from animal protection groups both at home and abroad [4]. How to balance the contradiction between traditional culture preservation and animal rights protection is a challenge ahead. Encouragement and support for the development of bear bile substitute, especially in terms of common livestock biles and traditional Chinese medicinal plants, may help solve this contradiction. As we mentioned above, artificial bear bile, which has been demonstrated to possess the potential to replace the use of bear bile several decades ago, still has not been approved for marketing so far. The reason for this justified by related department is puzzling and mainly involved profits. The approval of artificial bear bile may reduce the market demand on natural bear bile, thereby affecting the great profits made from bear bile industry. But no matter how, people should not sacrifice the innocent bears only for self-profits and ignore inhumanity and wild sources protection. Indeed, government has the responsibility to preserve national culture; therefore, supports seeking potential substitutes for bear bile rather than simply prohibition are preferred. In the future, administrative department should be more prudent in approving the bear bile derived product in order to reduce the usage of bear bile to a large extent. Furthermore, policy systems to protect and support the research and development of substitute of bear bile should be considered [6, 14].

### 5.3. Public, Media, and Other Organizations

In addition to scientists and government, public, media, and other organizations could also contribute to the realization of bear bile substitute. As of today, media is a powerful strength and we should take full advantage of it for the progress of bear bile substitute. In fact, it was the media that exposed the miserable lives of bears in the cage for bile extraction, which attracted wide attention from the public afterwards. A company named Gui Zhen Tang, which mainly runs business in bear industry, attempted to be a listed company and seems to get supports from government and Association of Chinese Medicine in 2012. By the intensive condemnation and protests from the public and mass media, finally, the review process was suspended in 2013. In this case, the public and media play a very important role in the protection of bear bile, which in turn promotes the development of bear bile substitute [4]. Apart from exposure, the media promotion of substitutes for bear bile to the public is of importance. To this day, plenty of people still have blind faith in rare and expensive traditional drugs due to history and traditional culture. People should get the point that getting bear bile from living bears failed to protect bears; conversely, they are subjected to incredible pain and suffering. Changing the mind of people to accept the substitute of bear bile is the first step, of course, which needs convincing scientific evidences to support [15]. Given that using bear bile is a part of Chinese culture and existing TCM practice, some people insist that, no matter how close those substitutes can be, they are still not as good as the real ones. Completely replacing real one in bear bile chemical compositions is really difficult, but pharmacological effect of the substitute which is better than real bear bile is possible and real. With regard to other related organizations, such as animal protection organization and Non-Government Organization, they are supposed to offer education, appropriate guidance, and surveillance to protect bears and publicize the promising substitutes.

## 6. Conclusion

In conclusion, the development of bear bile substitute depends on the collaboration and efforts of many aspects. The use of substitutes is a very prudent issue, which needs persuasive research data, strict examination, and quality control. Although increasing scientific evidences have demonstrated that artificial bear bile, several kinds of animal biles, synthetic compounds, and medicinal plants have the potential to substitute bear bile, it will take long time to stop the use of bear bile with lots of struggles and challenges. Overall, scientific comparative research, policy support, and promotion of the substitutes are rational and indispensable strategy to realize the replacement gradually in the future.

## Figures and Tables

**Table 1:** The presence of various bile salts in some animal biles [72–75].

Animal	Bile salts
Bear	TUDC, TC, TDC, TCDC
Dog	TC, TCDC, TDC
Cattle	TC, TDC, TCDC, TLC, GC, GDC, GCDC, GLC, AC
Pig	TC, TDC, TCDC, THC, THDC, GC, GDC, GCDC, GHC, GHDC, GHOC
Snake	TPC, TC, TDC
Rabbit	GDC, TC, TDC, TCDC
Chicken	TC, TCDC, TAC
Fish	TCDC, TC, TDC, AC
Goose	TCDC, TPHC, TBC
Duck	TCDC, TPHC, TBC
Mouse	TC, TDC, TCDC, T-*β*-MC, T-*ω*-MC, TUDC

TUDC: tauroursodeoxycholate; TC: taurocholate; TDC: taurodeoxycholate; TCDC: taurochenodeoxycholate; TLC: taurolithocholate; GC: glycocholate; GDC: glycodeoxycholate; GCDC: glycochenodeoxycholate; GLC: glycolithocholate; AC: allocholate; THC: taurohyocholate; THDC: taurohyodeoxycholate; GHC: glycohyocholate; GHDC: glycohyodeoxycholate; GHOC: glyco-3*α*-hydroxy-6-oxo-5*β*-cholate; TPC: tauropythocholate; TAC: tauroallocholate; TBC: taurobitocholate; TPHC: taurophocacholate; T-*β*-MC: tauro-*β*-muricholate; T-*ω*-MC: tauro-*ω*-muricholate.
